# Detection and Classification of Peri‐Implant Marginal Bone Loss in Cone‐Beam Computed Tomography Using a Deep Learning Approach

**DOI:** 10.1002/cre2.70308

**Published:** 2026-02-17

**Authors:** Zahra Madani, Hoorieh Bashizadeh Fakhar

**Affiliations:** ^1^ Department of Maxillofacial Radiology, Faculty of Dentistry Tehran University of Medical Sciences Tehran Iran

**Keywords:** cone‐beam computed tomography, deep learning, dental implants, peri‐implantitis

## Abstract

**Objectives:**

Modern dental implants have high long‐term survival, but peri‐implant marginal bone loss remains a multifactorial cause of implant failure and often is radiographically occult. Cone‐beam computed tomography (CBCT) provides superior 3D assessment but produces large datasets requiring expert interpretation. Deep‐learning object‐detection models like YOLOv8 may automate detection and grading. This study aimed to evaluate a YOLOv8‐based model for automated detection and grading of peri‐implant marginal bone loss on 2D images derived from CBCT.

**Materials and Methods:**

This retrospective study used 699 2D CBCT sections. Marginal bone loss was graded into four classes (≤ 20%, 21%–40%, 41%–60%, > 61% of implant length). A YOLOv8 detector was trained on 600 × 600‐pixel images (bounding box width 20 px) with two classes (implant, bone loss), split 80/10/10 (train/test/val), 200 epochs, batch size 8 and tuned hyperparameters. Performance was assessed by accuracy, precision, recall, and F1‐score.

**Results:**

The YOLOv8 model achieved strong diagnostic performance on the test set, with overall accuracy, precision, recall, and F1‐score of 0.90. It performed best for healthy sites (precision 0.92, recall 0.99, F1 0.95) and maintained high performance for mild lesions (F1 0.90), while moderate and severe cases showed reduced metrics (F1 0.62 and 0.70, respectively). A three‐class scheme had 0.88 accuracy and excellent reliability (Kappa = 0.954). Detection metric included mAP@0.5 (mean precision at intersection over union threshold of 0.5) of 0.889, a recall of 0.98 at low threshold, and implant‐length detection accuracy of 1.00. Training stabilized after about 15 epochs.

**Conclusions:**

The YOLOv8‐based deep learning model can reliably detect and grade peri‐implant marginal bone loss on CBCT images. Future research should expand datasets, incorporate multimodal information, and validate performance across diverse clinical settings.

## 1 Introduction

Advances in implant design and clinical protocols in modern dental implantology have led to long‐term survival and success rates exceeding 90% for dental implants (Howe et al. 2019). However, biological and mechanical complications remain important causes of late implant failure. Among biological complications, peri‐implantitis leading to marginal bone loss is a leading concern, with prevalence rates of peri‐implantitis reported as approximately 22% at the patient level (Schwarz et al. 2018) and 8.9% for advanced cases in a recent private practice study (Ciurescu et al. 2025), as it reduces osseointegration and can result in implant mobility or loss (Srinivasan et al. 2017). Peri‐implantitis is a multifactorial inflammatory disease primarily caused by bacterial colonization and plaque accumulation on the implant surface, with modifying factors such as poor oral hygiene, history of periodontitis, lack of keratinized mucosa < 2 mm, occlusal loading, implant design, cortical bone thickness, behavioral risk factors like smoking, systemic conditions like diabetes, and implant function time > 5 years influencing its onset, progression, and rate of associated bone resorption around implants (Schwarz et al. 2018; Ciurescu et al. 2025; Şener‐Yamaner et al. 2017; Romandini et al. 2021). Early stages of marginal bone loss (resulting from peri‐implantitis) can be asymptomatic, so that periodic radiographic monitoring and sensitive detection methods are essential for timely intervention to preserve implant prognosis (Bornes et al. 2023).

Conventional two‐dimensional (2D) intraoral and panoramic radiography has been the main method for routine peri‐implant assessment, but is limited by projectional distortion, anatomical superimposition, and variable image geometry that can obscure true 3D bone defects around implants (Pauwels et al. 2015). Cone‐beam computed tomography (CBCT) provides volumetric, high‐resolution views of buccal, lingual, and proximal bone plates, and has been shown to improve detection, classification and measurement of peri‐implant bone defects compared with intraoral imaging, making CBCT an effective tool for assessing of peri‐implant bone status (Gholampour et al. 2024). CBCT provides more anatomical information that can facilitate more accurate mapping of defect morphology and depth, though detection accuracy can be influenced by factors such as buccal bone plate thickness, implant‐abutment connections (e.g., zirconia–zirconia interfaces), and the number of implants in the field of view (Liedke et al. 2017; Domic et al. 2021). While clinical protocols should prioritize a minimized field of view to adhere to as low as reasonably achievable (ALARA) principles and reduce radiation exposure, the resulting large volumetric datasets require specialized expertise and time‐consuming interpretation by experienced clinicians (Song et al. 2021).

Artificial intelligence (AI), and particularly deep learning using convolutional neural networks (CNNs) and modern object‐detection architectures (e.g., the YOLO family), have demonstrated strong potential for automated interpretation of medical and dental images, improving detection accuracy and reducing observer variability in a range of tasks (Lee et al. 2024; Vera et al. 2023). Deep learning models can identify caries, periodontal bone loss, and periapical lesions from panoramic radiographs and CBCT scans, offering a significant advantage in efficiency and consistency compared to traditional manual assessments (Ryu et al. 2023).

Recent dental imaging studies have used deep‐learning models to detect implants and peri‐implant bone changes on 2D radiographs and have reported promising diagnostic performance. However, most used periapical or panoramic images and relatively limited datasets or implant systems (Liu et al. 2022; Cha et al. 2021; Chen et al. 2023). A number of studies have implemented object‐detection or region‐based CNN approaches to localize implants and quantify marginal bone loss with favorable metrics, but these have limitations such as variability in annotation protocols and limited external validation across implant types and imaging modalities (Cha et al. 2021; Chen et al. 2023; Chang et al. 2020). Given CBCT's higher anatomic details and speed improvements in newer YOLO versions, the evaluation of advanced YOLO‐based deep‐learning algorithms for automated detection and grading of peri‐implant marginal bone loss on 2D sections from CBCT datasets is scarce (Lee et al. 2024). Therefore, this study aimed to evaluate the performance of a YOLOv8‐based deep learning model for automated detection and grading of marginal bone loss around dental implants on 2D CBCT images, comparing model outputs with expert radiologist assessment and standard diagnostic metrics.

## 2 Methods

### 2.1 Study Design and Ethical Considerations

This retrospective study was approved by the Ethics Committee of the Tehran University of Medical Sciences. All research was conducted in accordance with relevant guidelines and regulations (World Medical Association 2013; Collins et al. 2024). The reporting of this study adheres to the TRIPOD + AI statement for transparency in AI‐based clinical prediction models (Collins et al. 2024). In our study, the requirement for informed consent was waived by the Ethics Committee of the Tehran University of Medical Sciences, as the research involved retrospective analysis of anonymized CBCT images. The study protocol was reviewed and approved under approval number IR.TUMS.DENTISTRY.REC.1403.059.

### 2.2 Study Population, Data Collection, and Preparation

A dataset of 1205 2D CBCT sections was randomly collected from the archives of a private oral and maxillofacial radiology practice to standardize diagnostic quality. All CBCT volumes were acquired using a Planmeca ProMax 3D Max device (Planmeca Oy, Helsinki, Finland) with the following parameters: 92 kV, 10 mA, scan time 12 s, voxel size 200 μm, and field of view 100 × 90 mm. The images were then examined and measured using Planmeca Romexis software (version 2.9.2.R, Planmeca Oy, Helsinki, Finland). The filtering was conducted through manual review against inclusion and exclusion criteria by the primary evaluator (H.B.F.) and assistant (Z.M.). Approximately 506 sections were excluded: 250 due to high‐degree artifacts impeding diagnosis, 150 for anterior implants, 80 for incomplete follow‐up records, and 26 for bone grafting history. After filtering, a total of 699 2D CBCT sections (derived from 186 implants in 94 patients) were included in the study.

The initial views of CBCT were reconstructed in both coronal and sagittal planes, centered on the implant. A region of interest was selected based on the implant diameter. For each implant, pseudo‐panoramic images were reconstructed to evaluate the mesial and distal aspects, with the panoramic line drawn perpendicular to the long axis of the implant. Furthermore, 2–4 cross‐sectional images were generated (chosen to sufficiently capture buccal and lingual dimensions while minimizing unnecessary slices, influenced by defect visualization needs and data efficiency) with a minimum slice thickness of 0.3 mm and a slice interval of 1 mm to examine the buccal and lingual dimensions. To standardize the pixel count, the images were arranged on the software's defined print layout.

Inclusion criteria were as follows: (1) patients over 18 years of age; (2) presence of bone‐level dental implants that had been prosthetically loaded; (3) CBCT images taken during follow‐up; (4) implants placed in posterior regions only. Exclusion criteria included: (1) complete implant failure; (2) incomplete periodontal follow‐up or missing records; (3) history of bone grafting procedures; (4) images with a high degree of artifacts that impeded diagnosis of marginal bone levels.

After saving the unlabeled images, all sections of the implants were evaluated by an oral and maxillofacial radiology specialist with over 30 years of clinical experience (H.B.F., primary evaluator, along with an assistant [Z.M.]). Discrepancies in measurements were initially resolved by consensus. If necessary, the radiographs were re‐evaluated by a third observer. Areas of bone loss and implant length were marked and measured in millimeters using the software's measurement tools. A horizontal reference line was first drawn at the abutment‐implant border. Then, lines for marginal bone loss were drawn perpendicular to this reference line, extending to the most apical point of bone loss observed on the image. The implant length was measured as the vertical distance from the horizontal line to the most apical part of the implant at its center. The ratio of the bone loss length to the implant length was used to grade the bone loss. Images were oriented with the implant crown upward for consistency in analysis across maxillary and mandibular implants.

We defined four distinct grades for the classification of peri‐implant marginal bone loss, with borderline cases assigned based on the calculated numerical ratio without rounding (e.g., 20.0% as Grade 1, 20.1% as Grade 2): Grade 1 for bone loss less than or equal to 20% of the implant length, Grade 2 for bone loss between 21% and 40%, and Grade 3 for bone loss ranging from 41% to 60%. Grade 4 was assigned to cases with bone loss greater than 61% of the implant length. Grading was performed per site (buccal, lingual, mesial, distal) based on the specific view, allowing for varying grades across aspects of the same implant.

We used YOLOv8 (Ultralytics 2023), the latest version in the “You Only Look Once” series. The architectural elements include a modified Stem with a smaller convolutional kernel (transitioning from 6 × 6 to 3 × 3) and a new Backbone structure that uses the cross‐stage partial with 2 convolutions (c2f) module, which improves feature representation. Furthermore, YOLOv8 uses mosaic augmentation during training, combining four images to enhance object detection under various conditions.

### 2.3 Training Configuration and Evaluation

In the pre‐processing stage, all images were resized to a fixed size of 600 × 600 pixels. This resolution was chosen after evaluating pixel dimensions of 512, 600, 640, and 800, as it showed the best results. Based on the lines marked by the specialist, a bounding box with a width of 20 pixels was used to define the area for both bone loss and the implant. Images were processed and loaded using a custom Python script with the Ultralytics YOLO library: preprocessing via OpenCV for resizing, annotation files generated in YOLO format (.txt with normalized coordinates), and data loaded via PyTorch DataLoaders with augmentations applied.

Then, two classes were defined: class zero for implants and class one for bone loss. The implant location was defined by a midpoint line, with bone loss lines placed to the left and right of the implant. Both were assigned to the bone loss class. For each image, the bounding box was identified, and if it was larger than 14 pixels, it was considered a new annotation box, and its length, width, and height were saved. A total of 699 images were reviewed and annotated in this manner. The model's task was to identify the 20‐pixel region around each marked line. The 20‐pixel width was selected through trial and error to optimize model performance.

The images were categorized into three sets: 80% for training (559 images), 10% for testing (70 images), and 10% for validation (70 images). The model was trained for 200 epochs to ensure it effectively learned the features necessary for detecting bone loss and implant length. A batch size of eight was used. The initial learning rate was set to 0.01. These parameters were optimized through hyperparameter tuning, which involved 45 different evaluations to achieve the best‐performing model.

The performance was evaluated using standard metrics, including accuracy (the ratio of correct predictions to the total number of samples), precision (the proportion of positive predictions that were actually correct), recall (the ability to correctly identify all relevant instances), and F1‐score (a balanced measure of the model's ability to accurately and completely identify the target features). Statistical analysis involved computing these metrics using scikit‐learn in Python on test set predictions, with Cohen's Kappa for classification agreement.

## 3 Results

**Figure 1:**
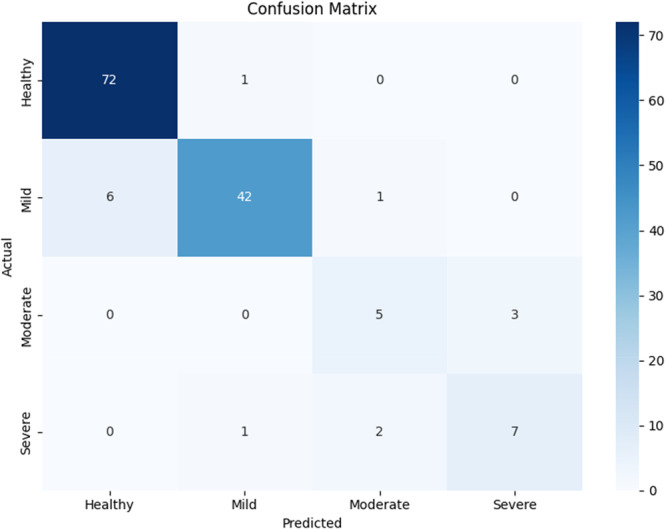
Confusion matrix illustrating the model's classification performance across all grades of bone loss and background. The rows of the matrix represent the model's predicted labels, while the columns correspond to the true labels.

The model demonstrated strong overall performance with an overall accuracy of 0.90, and weighted average precision, recall, and F1‐score of 0.80, 0.79, and 0.80, respectively (Table 1). The model was most effective at identifying healthy cases, with a precision of 0.92, recall of 0.99, and an F1‐score of 0.95. As the severity increased, the model's performance decreased, with moderate and severe cases showing F1‐score of 0.62 and 0.70, respectively (Table 1 and Figure 1).

**Table 1:** Performance of the model in detecting and classifying bone loss into four grades.

Class	Precision	Recall	F1‐score	Number of instances per class in the test set
Healthy	0.92	0.99	0.95	73
Mild	0.95	0.86	0.90	49
Moderate	0.62	0.62	0.62	8
Severe	0.70	0.70	0.70	10
Accuracy	0.90			140
Macro avg	0.80	0.79	0.80	140
Weighted avg	0.90	0.90	0.90	140

When mapped to the traditional three‐class classification system (Monje et al. 2019), excluding healthy cases, our classification showed an overall accuracy of 0.88, with a high F1‐score for mild cases (0.91), and lower performance for moderate (0.79), and severe (0.86) cases. The reliability of the data for this analysis was assessed using a Kappa index, which was found to be 0.954, indicating excellent agreement (Table 2).

**Table 2:** Performance of the model in detecting and classifying bone loss into three grades.

Class	Precision	Recall	F1‐score	Number of instances per class in the test set
Mild	0.88	0.95	0.91	86
Moderate	0.85	0.74	0.79	39
Severe	0.80	0.92	0.86	15
Accuracy	0.88			140
Macro avg	0.84	0.87	0.85	140
Weighted avg	0.86	0.89	0.87	140

**Figure 2:**
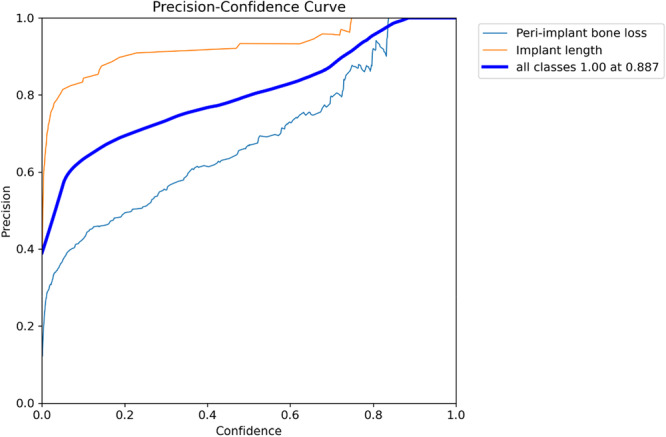
Precision‐confidence curve for bounding box predictions. The curve shows the model's ability to maintain high precision at various confidence thresholds.

**Figure 3:**
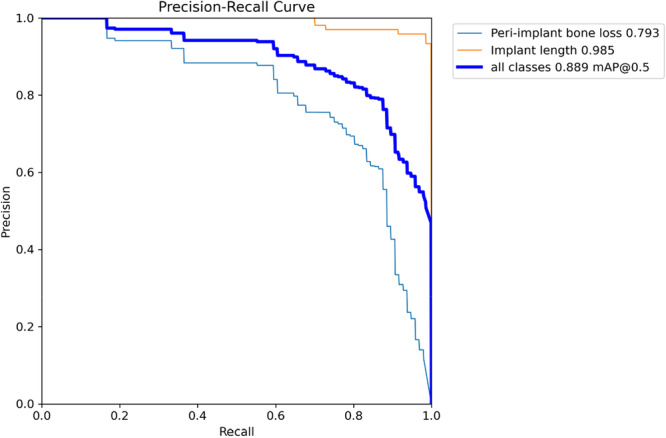
Precision‐recall curve for bounding box predictions. This figure demonstrates the model's effectiveness in balancing precision and recall.

**Figure 4:**
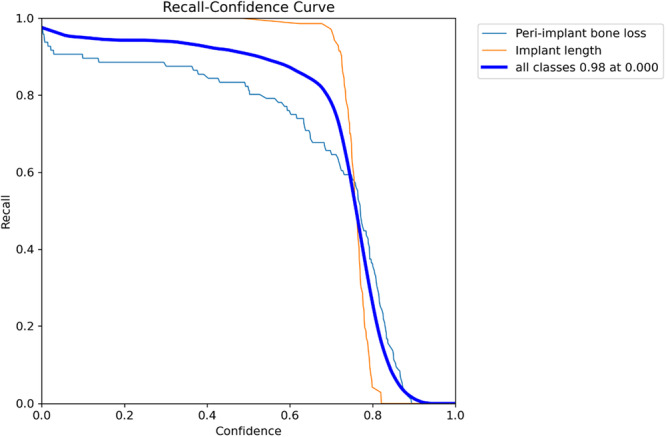
Recall‐confidence curve for bounding box predictions. This curve shows the model's high sensitivity in detecting true positive cases.

**Figure 5:**
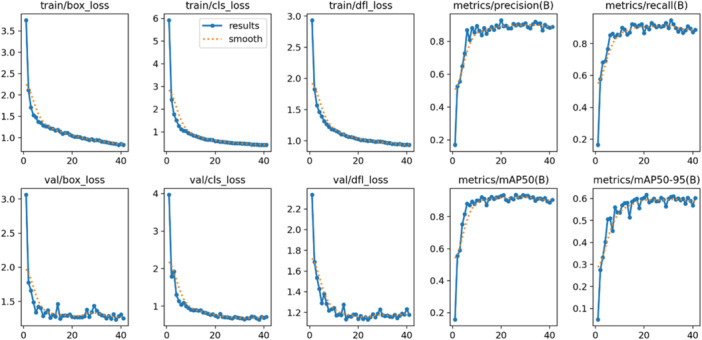
Training and validation loss curves and metrics. The figure illustrates the model's learning process and the improvement of key performance metrics over time.

**Figure 6:**
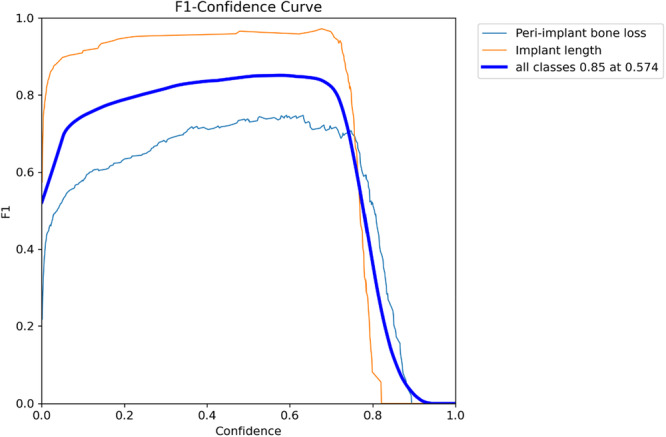
F1‐confidence curve for bounding box predictions. The curve demonstrates the model's balanced performance at various confidence thresholds.

Regarding diagnostic performance, the precision‐confidence curve showed that the model maintained high precision, with implant length detection reaching 1.00 precision at a confidence of 0.887 (Figure 2). The precision‐recall curve demonstrated an overall average precision (mean precision at intersection over union threshold of 0.5; mAP@0.5) of 0.889, showing the model's high overall capability in detection (Figure 3). The recall‐confidence curve showed a high overall recall of 0.98 at a confidence threshold of 0, indicating the model's high sensitivity in identifying true positive cases (Figure 4). Analysis of the training and validation loss curves confirmed that the model learned progressively and optimally, with the loss and performance metrics stabilizing after approximately 15 epochs (Figure 5). The F1‐confidence curve showed a balanced performance, with an overall F1‐score of 0.85 at a confidence level of 0.574 (Figure 6).

**Figure 7:**
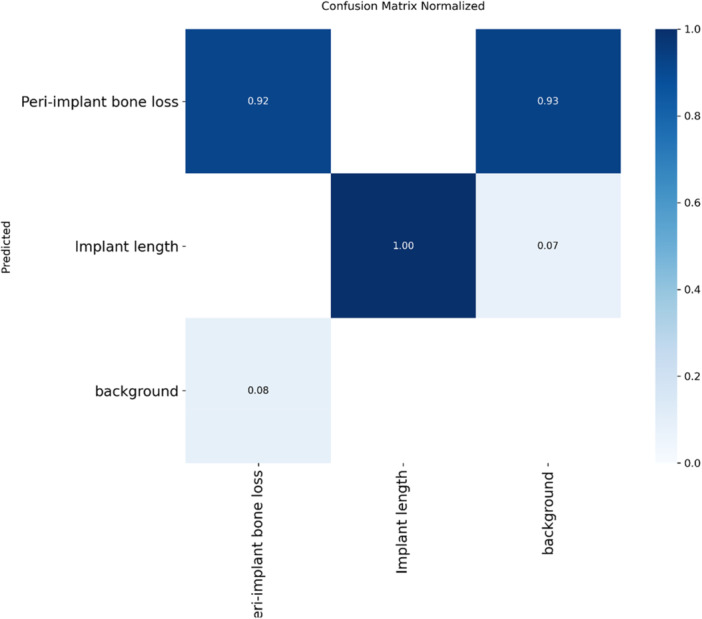
Normalized confusion matrix for the classification of bone loss, implant length, and background.

**Figure 8:**
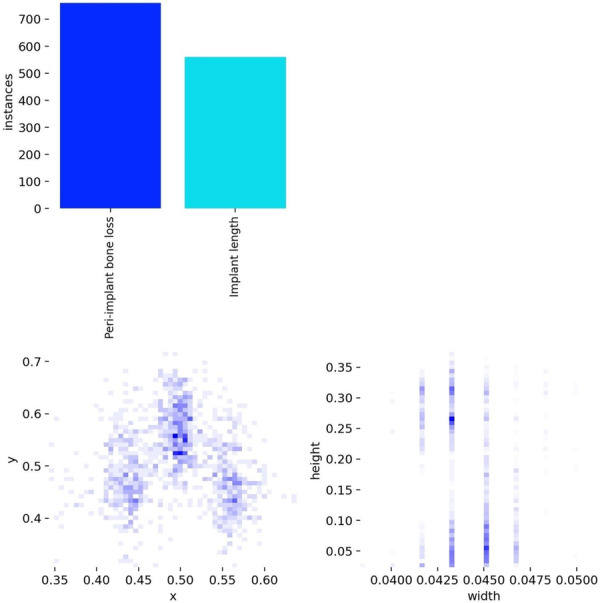
Distribution of labels in the dataset. This figure highlights the distribution of “bone loss” and “implant length” instances, showing the composition of the dataset.

The model's performance in detecting implant length was perfectly accurate at 1.00, and 0.92 of the true positive cases for bone loss were correctly identified (Figure 7). The distribution of annotated labels showed that the dataset was well‐balanced, with approximately 700 instances of bone loss and 550 instances of implant length (Figure 8).

Figures S1 and S2 provide visual examples of the model's segmentation results, showing its ability to accurately delineate both bone loss and implant length.

## 4 Discussion

Our study showed that a modern object‐detection deep‐learning architecture can automatically identify and categorize marginal peri‐implant bone loss on 2D sections from CBCT volumes with high overall reliability, particularly in distinguishing healthy sites and mild bone resorption from more advanced defects. This finding highlights the potential of combining volumetric imaging with efficient detection models to support routine radiologic screening and diagnostic workflows in implant dentistry. YOLOv8 is a type of AI known as a deep learning model, which falls under subsymbolic AI and machine learning principles. It works like a smart pattern recognizer. It learns to spot objects by analyzing thousands of example images, using layers of mathematical calculations to identify features like edges and shapes, rather than following explicit programmed rules. This makes it fast and effective for tasks like real‐time detection in medical imaging.

Our model performed best for healthy sites and mild bone loss, with progressively reduced metrics for moderate and severe cases. This suggests that it might be more suitable for screening and early‐warning applications. Similar studies that applied region‐based or CNN‐based approaches to periapical radiographs also reported good performance for detecting the presence or absence of marginal bone loss and for identifying early lesions, supporting the idea that deep networks capture discriminative radiographic features of subtle bone changes. In this regard, Liu et al. found that a Faster R‐CNN approach on periapical films provided promising agreement with expert readers for detecting marginal bone loss, especially for clear, well‐defined lesions. It reported Kappa values equal to 0.547 for bone loss sites and 0.568 for bone loss implants (Liu et al. 2022). Furthermore, Chen and colleagues showed that CNN‐based systems can measure peri‐implant damage on periapical images with clinically useful accuracy (approximately 90%), highlighting the utility of automated tools for early detection and standardized reporting (Chen et al. 2023). Recent reviews of AI in implant radiology also reported that models tend to perform best when distinguishing absence of disease from mild disease in well‐annotated datasets (Ibraheem 2024).

Compared to other deep learning models used in dental radiography, YOLOv8 has advantages in speed and efficiency for object detection tasks. For instance, studies have shown YOLO variants outperforming Mask R‐CNN (e.g., mean average precision at 0.5 intersection over union of 98.9% vs. 95%) and Faster R‐CNN in accuracy and inference time for detecting dental structures or pathologies, such as caries or root resorption (Çetinkaya et al. 2025). Similarly, YOLO models generalize better with limited data and lower‐resolution images compared to models like Single Shot Detector or Detectron2, making them suitable for real‐time clinical applications in implant assessment (Vilcapoma et al. 2024).

On the other hand, the reduced performance of our model for moderate and severe categories aligns with prior work and likely reflects a combination of class imbalance, increased heterogeneity of severe defect morphology, and annotation uncertainty. Although mild bone loss is often clinically more challenging to detect due to its subtlety, our model performed better on these cases possibly because they were more represented in the dataset, allowing the algorithm to learn consistent early‐stage features. In contrast, moderate and severe losses showed greater variability in defect shapes, compounded by potential imaging artifacts or overlapping structures, which confused the model despite their apparent visibility (Mao et al. 2025). Class imbalance has commonly been identified as a cause of lower recall and precision for less frequent classes in dental imaging studies and can bias learning toward the majority classes (Ameli et al. 2024). Morphological overlap between deep crater‐type defects and vertical loss, and the effects of metal artifacts or CBCT reconstruction variability, make graded distinctions harder to learn from single‐slice or limited‐view reconstructions. Moreover, similar challenges were noted in studies that compared 2D and 3D imaging, as well as those addressing segmentation and staging, where lesion heterogeneity and imaging noise degrade classifier separability (Ameli et al. 2024; Costa et al. 2023). Practical remedies reported in the literature include data‐level balancing, targeted augmentation for minority classes, incorporation of multiplanar/volumetric inputs, and hybrid detection‐segmentation pipelines; which align with recent best practices and might improve performance on moderate/severe categories in the future (Ameli et al. 2024; Erturk et al. 2025).

Our segmentation and detection metrics, showing high mAP@0.5 and stable precision–recall curves at optimized thresholds, indicate that an object‐detection approach like YOLOv8 can provide both fast inference and spatial localization typically adequate for clinical overlays. This aligns with recent applications of YOLO‐family models and other lightweight detectors to dental radiography where rapid, robust localization was achievable without extreme computational demands (Park et al. 2023; Bonfanti‐Gris et al. 2025). The model provides bounding information that directly guides the clinician's attention and supports near real‐time inference that can be integrated into imaging workstations. Prior studies aiming at precise quantification has combined detection with specialized segmentation networks or 3D segmentation frameworks to estimate defect volume or linear dimensions (Ameli et al. 2024). Integrating a segmentation stage, such as no new U‐net (nnU‐Net) or a 3D U‐Net variant, with YOLO‐style detection might therefore be a next step to produce actionable numeric measures for surgical planning (Ameli et al. 2024).

Our study has several clinical implications. A tool that reliably flags healthy versus early bone loss on 2D CBCT images could support routine monitoring by reducing oversight of subtle changes, standardizing longitudinal comparisons, and prompting timely interventions such as localized debridement or earlier regenerative measures, which may improve implant survival and patient outcomes (Al‐Asali et al. 2024). Moreover, the speed and consistency of an automated detector can reduce inter‐observer variability, free clinician time for higher‐level decision‐making, and enable triage workflows in high‐volume practices or tertiary referral centers (Albano et al. 2024). Furthermore, AI systems that augment clinician assessment can facilitate personalized surveillance schedules and targeted treatment planning, particularly when combined in the future with clinical and demographic data to form a multimodal risk model (Gao 2024; Huang et al. 2022).

Our strengths include the use of 2D sections from CBCT volumes rather than only 2D projections, though we acknowledge that the final analysis and measurements were performed on reconstructed 2D sections derived from the volumes, which is standard but may not capture full 3D information. The application of a computationally efficient detection architecture suitable for near real‐time use, and comparison of model outputs to expert radiologist labels using multiple performance metrics. However, there are limitations that must be acknowledged. First, the dataset showed class imbalance with relatively few moderate and severe cases, which likely reduced per‐class performance and limits conclusions about advanced defects. Second, the labeling protocol relied on manual annotations that can vary between readers and thus might introduce uncertainty. Third, the study used a limited number of scanner settings and an institutional population, so external validation on diverse CBCT hardware, implant systems and patient populations is required to establish broad clinical utility. Additionally, CBCT imaging has inherent limitations in detecting peri‐implant defects, including sensitivity to voxel size (smaller voxels improve resolution but increase noise), field of view (larger field of view may reduce detail), implant‐related metal artifacts (e.g., beam hardening from titanium), and buccal bone thickness (thinner plates < 1 mm are harder to visualize) (Liedke et al. 2017; Domic et al. 2021). Our dataset did not include detailed information on supra‐structures (e.g., crowns, abutments) or specific implant materials, which could influence artifact levels and detection accuracy but were not accounted for. Furthermore, we excluded scans with high‐degree metal artifacts to ensure annotation quality, which may limit the model's applicability in real‐world clinical settings where such artifacts are prevalent and could degrade performance.

## 5 Conclusions

We demonstrated that a YOLOv8‐based deep learning model can automatically detect and grade peri‐implant marginal bone loss on 2D CBCT images with high overall reliability, particularly at distinguishing healthy sites and mild bone resorption. Its fast inference and localization can make it an assistive tool for radiologic screening and clinician decision support. Future studies should expand the dataset with multicenter CBCT scans and a greater number of moderate and severe cases to improve performance, provide precise quantitative measurements, and explore multimodal learning that combines imaging with demographic and clinical variables to improve prognostic accuracy. Furthermore, external validation across different scanners and implant systems, assessment of real‐world clinical workflow integration and user acceptability, prospective studies to evaluate impact on patient outcomes, and efforts to improve model explainability will be important steps toward clinical translation.

## Author Contributions

Conceptualization and study design: Zahra Madani and Hoorieh Bashizadeh Fakhar. Data acquisition: Zahra Madani and Hoorieh Bashizadeh Fakhar. Data analysis/interpretation: Zahra Madani and Hoorieh Bashizadeh Fakhar. Statistical analysis and coding of the AI model: Zahra Madani. Supervision/mentorship: Hoorieh Bashizadeh Fakhar. Drafting of the manuscript: Zahra Madani and Hoorieh Bashizadeh Fakhar. Responsibility of submitting for publication: Zahra Madani. All authors had a critical role in revision of the manuscript for important intellectual content. The final manuscript was read and approved by all authors.

## Funding

The authors received no specific funding for this work.

## Ethics Statement

This retrospective study was approved by the Ethics Committee of the Tehran University of Medical Sciences (Ethics Code: IR.TUMS.DENTISTRY.REC.1403.059). All research was conducted in accordance with relevant guidelines and regulations. Informed consent was not required, as the study utilized archived, de‐identified CBCT radiographic images, with no access to patient demographic or clinical information.

## Consent

The requirement for informed consent was waived by the Ethics Committee of the Tehran University of Medical Sciences, as the research involved retrospective analysis of anonymized CBCT images. The study protocol was reviewed and approved under approval number IR.TUMS.DENTISTRY.REC.1403.059. We confirm that all procedures complied with applicable national regulations and institutional guidelines.

## Conflicts of Interest

The authors declare no conflicts of interest.

## Supporting information


**Figure S1:** An example of the segmentation results for implant length and bone loss regions. **Figure S2:** Another example of the segmentation results for implant length and bone loss regions.

## Data Availability

The data that support the findings of this study are available on request from the corresponding author. The data are not publicly available due to privacy or ethical restrictions.
