# Supplementary material for: Detection and Classification of Peri‐Implant Marginal Bone Loss in Cone‐Beam Computed Tomography Using a Deep Learning Approach
Source: Clin Exp Dent Res. 2026 Feb 17;12(2):e70308. doi: 10.1002/cre2.70308 (PMC12914138; doi:10.1002/cre2.70308)
Supplement: Supplementary file 1 — Figure S1: An example of the segmentation results for implant length and bone loss regions. Figure S2: Another example of the segmentation results for implant length and bone loss regions. [file CRE2-12-e70308-s001.docx]

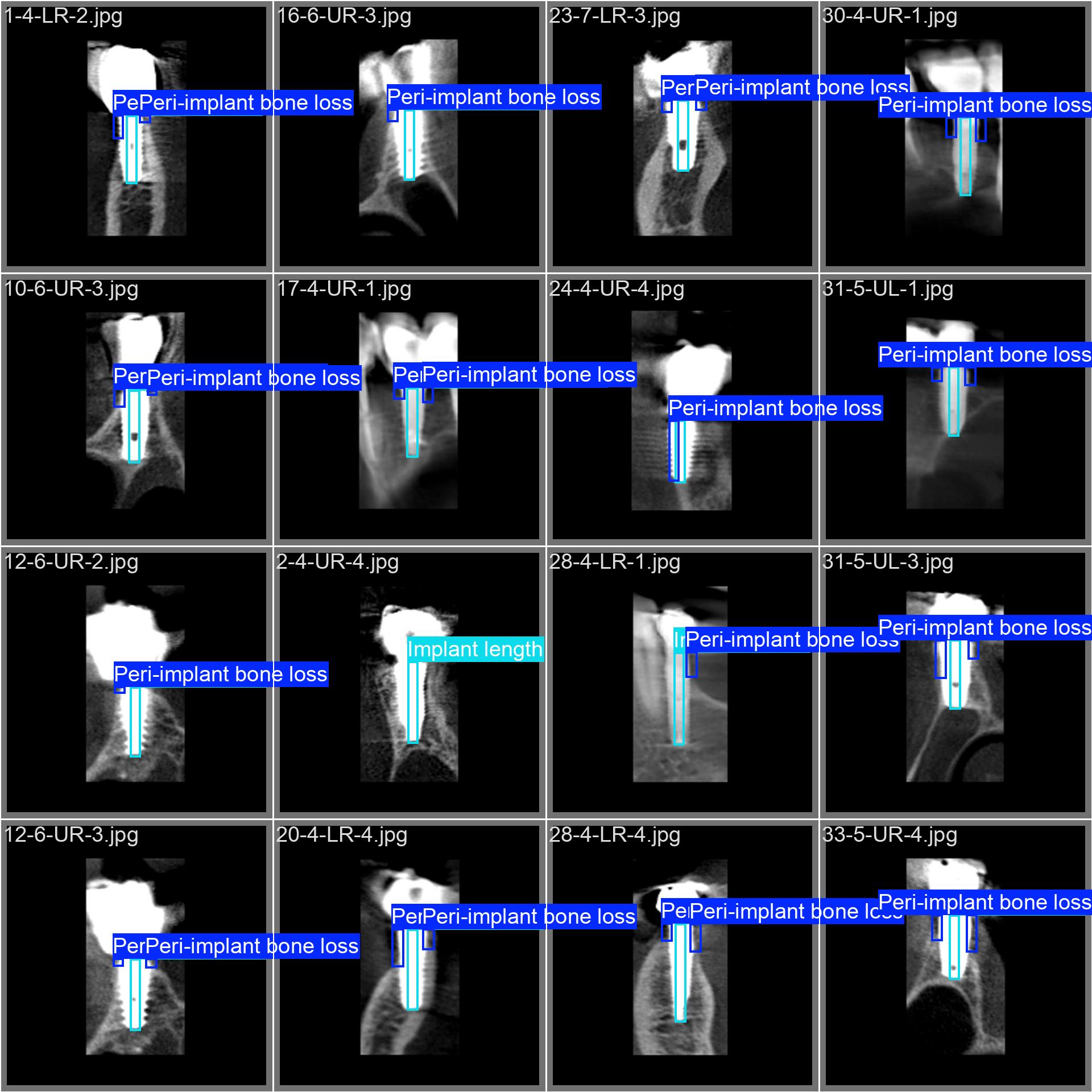


**Figure S1.** An example of the segmentation results for implant length and bone loss regions.

**
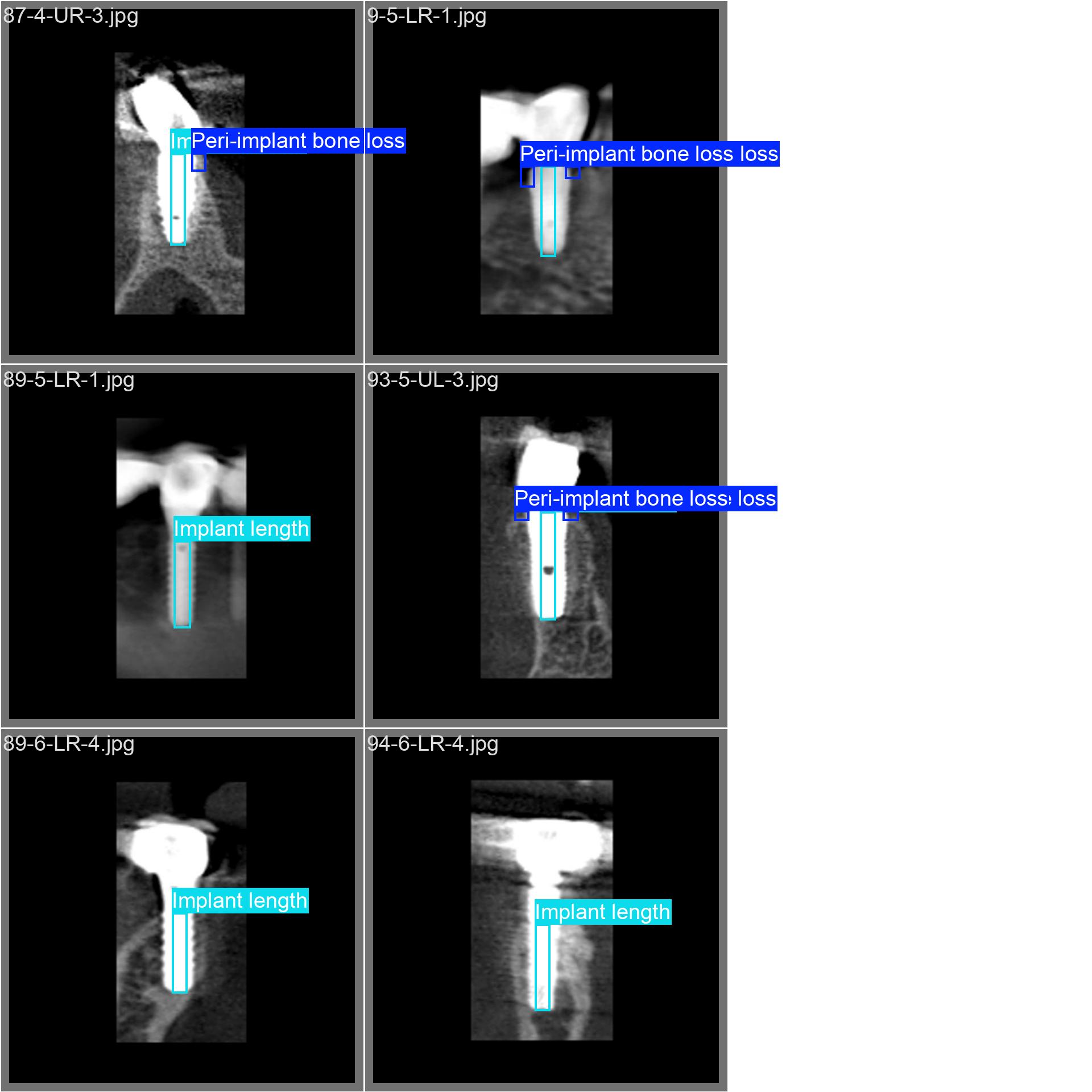
**

**Figure S2.** Another example of the segmentation results for implant length and bone loss regions.
